# Osgood-Schlatter Disease Unveiled Under High-frequency Ultrasonogram

**DOI:** 10.7759/cureus.3411

**Published:** 2018-10-04

**Authors:** Md Abu B Siddiq

**Affiliations:** 1 Physical Medicine and Rheumatology, Brahmanbaria Medical College, Brahmanbaria, BGD

**Keywords:** osgood schlatter disease, diagnosis, anterior knee pain, high-frequency ultrasonogram, musculoskeletal

## Abstract

Osgood-Schlatter disease is a painful condition, affecting the tibial tuberosity of physically active children with a painful bump that aggravates with repetitive impacts over the affected area during exercise, sports, or even usual daily activities. The condition is usually unilateral; however, bilateral presentation is not unlikely. In addition to clinical features, a unique radiological finding appears worthy in terms of accurate diagnosis. Moreover, ultrasonogram-based pathological changes could appear not only in the tibial tuberosity but also in the patellar tendon. In this study subject, I approached the condition with a high-frequency musculoskeletal ultrasonogram in order to add further information about the ailment in medical literature. The conservative approach was found effective to relieve the patient's problems.

## Introduction

Osgood-Schlatter disease (OSD) or osteochondrosis is a repetitive traction injury at the attachment of the patellar tendon to tibial tuberosity, causing an avulsion of tibial prominence from the tibial head [1]. It is one of the common causes of anterior knee pain among adolescents. Unilateral or bilateral usage anterior knee pain usually during the activities of daily living and or sports activity with a painful bump at the tibial tuberosity is the most common presentation; however, the patient could be asymptomatic. Since the knee joint vicinity remains unaffected, there is no limited range of motion. OSD is self-limiting and after 16 and 14 years, boys and girls, respectively, will no longer feel any pain [1]. Nonsteroidal anti-inflammatory agents (NSAIDs), ice/moist hot compression, local ultrasound therapy, etc. could be effective, though avoiding contact sports has been proved to be most appropriate [2]. The diagnosis of OSD is clinical and radiological - characteristic fragmented radio-opaque bone mass over the tibial prominence on X-ray [3]. The role of MRI and high-frequency diagnostic ultrasonogram (USG) in diagnosing OSD is not well depicted, though they appear potential when the pathology is not clearly delineated. Here, in this particular case study, I address the potential of cost-effective USGs in diagnosing OSD.

## Case presentation

**Figure 1:**
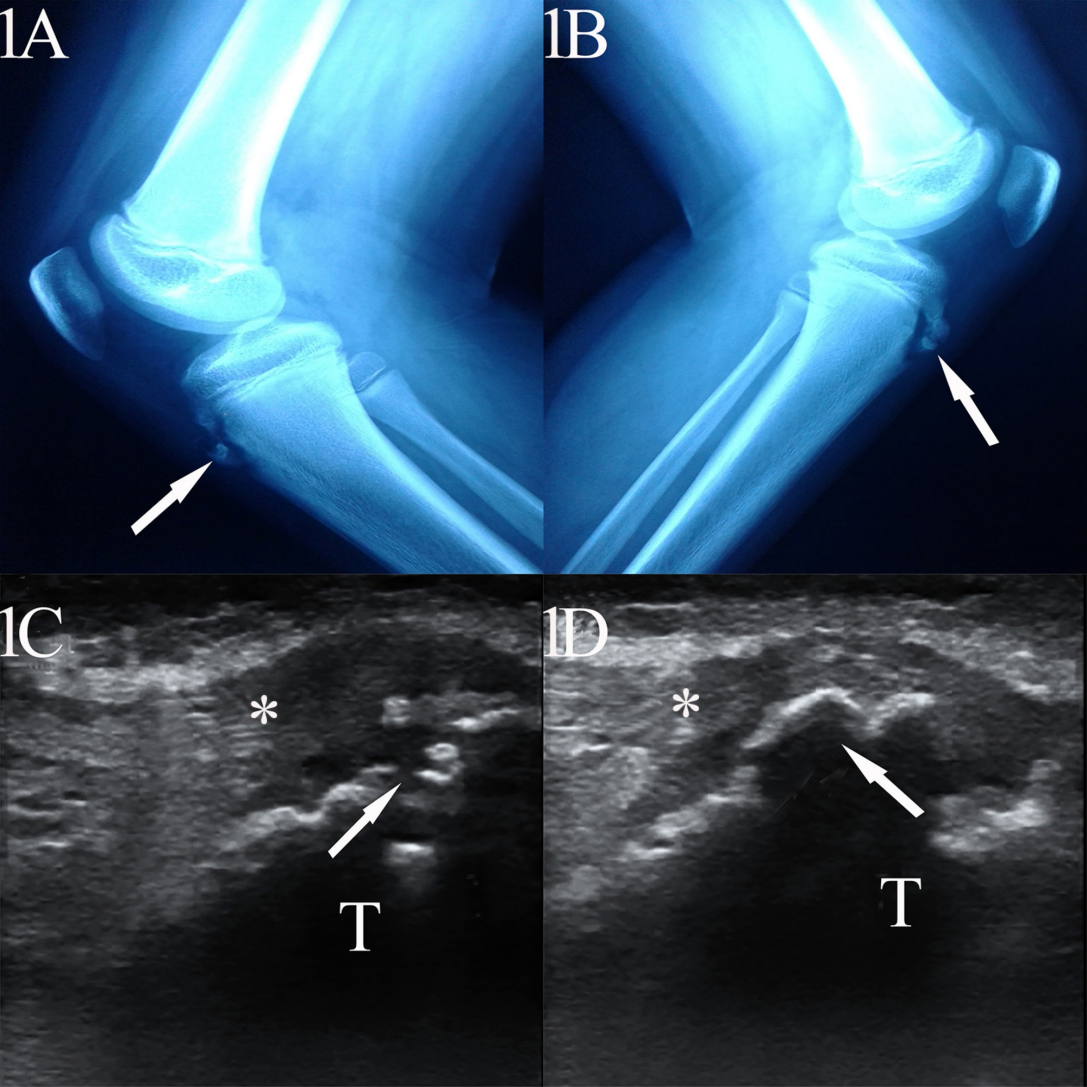
X-ray and ultrasonographic findings of Osgood-Schlatter disease 1A-1B: X-ray right and left knee, respectively, depict radio-opaque (arrow), fragmented mass over tibial prominences; 1C-1D: high-frequency linear probe ultrasonogram (longitudinal) of the right and left knee, respectively, illustrate a hyperechoic lesion surrounded with a hypoechoic lesion of unossified cartilage with a hypoechoic-thickened distal patellar tendon (asterisk)

A 12-year-old Asian-Bangladeshi boy presented with the complaint of pain in both anterior knees for three months. The pain aggravated while participating not only in contact sports but also with rapid walking, running, and kneeling. The physical examination revealed focal, swollen, tender areas over both knees; x-rays also documented a radiopaque, fragmented mass over the tibial prominences (Figures 1A-1B). An extended examination of the painful area under high-frequency (10 MHz) ultrasonogram with a linear probe (Chison ECO1, Jiangsu, China, 214142) unveiled a hyperechoic lesion surrounded with a hypoechoic lesion of unossified cartilage with a hypoechoic-thickened distal patellar tendon (Figures 1C-1D). There was no joint swelling and history of fever, malaise, and weight loss, and the nocturnal rise in body temperature and preceding localized knee trauma were also insignificant. All the aforementioned information is enough for diagnosing Osgood-Schlatter disease (OSD) in the present study. The patient was treated with oral diclofenac preparation (50 mg two times per day for three weeks). He was also advised not to participate in contact sports and, eventually, the patient was found pain free at his three-month follow-up.

## Discussion

Osgood-Schlatter disease is named after Robert Bayley Osgood, an American orthopedic surgeon, and Carl B. Schlatter, a Swiss surgeon, who described the condition independently in 1903 [1]. OSD can affect boys and girls around puberty. Boys between 10 and 15 years usually get the disorder unilaterally but a bilateral presentation can be seen in 20%-30% cases as well [3]. The condition could be asymptomatic though physical activity, including contact sports, repeatedly impacting the tibial tuberosity proliferates clinical manifestations suggestive of the disorder. On clinical examination, the tibial tuberosity appears swollen and tender on palpation, leaving no defect in the ipsilateral knee joint. A radiological investigation reveals dense radio-opaque bone fragments at the level of the tibial prominence with soft tissue swelling, which is unique for this childhood osteochondrosis. However, the condition is a diagnostic confusion with some other anterior knee disorders, for example, patella-femoral pain syndrome (PFPS) (chondromalacia patellae), jumper’s knee, Sinding-Larsen Johannssen syndrome, pre-patellar bursitis (housemaid’s knee), clergyman’s knee, parson’s knee (deep infra-patellar bursitis), anserine bursitis, etc. and, hence, it is required to exclude them while confirming OSD [4-10].

In PFPS, patients’ experience retropatellar pain, which is caused by compressive forces in the patella-femoral joint while climbing or descending stairs, squatting, and standing or sitting for a long time with flexed knees [4]. PFPS seems to be multifactorial and may result from the complex interactions between some factors that are intrinsic to joint anatomy and external training factors as well. PFPS is a common cause of knee pain in physically active adolescents and young adults, but it can occur at any age. The condition is often bilateral, but a unilateral presentation can be seen in clinical practice. Jumper's knee or patellar tendinopathy is an overuse injury due to repeated movements causing damage to the attachment of the patellar tendon to the tibial tuberosity, which also causes anterior knee pain [5]. Repeated jumping, landing, and changing direction result in strains, tears, and damage to the patellar tendon. So, children who regularly participate in sports involving repetitive jumping in track and field, basketball, volleyball, gymnastics, running, and soccer could experience the condition more. Common symptoms related to jumper's knee include pain directly over the patellar tendon, knee stiffness especially while jumping, kneeling, squatting, sitting, or climbing stairs, pain during knee bending, pain in the quadriceps muscle, leg or calf weakness, and focal tenderness at the patellar tendon insertion [4].

Sinding-Larsen Johansson disease, first described in 1921, is one of the reasons for childhood anterior knee pain and is seen more in boys aged between 12 and 14 years of age. Activity-related pain over the inferior pole of the patella is typical for the condition and is thought to result from persistent repetitive traction force by the patellar tendon on the lower pole of the patella and often cause a diagnostic confusion with the OSD [6-7].

Among various knee bursa, the pre-patellar bursa is the most commonly involved with the following clinical manifestations: knee pain, focal swelling, difficulty in kneeling, walking, and local tenderness. However, focal erythema raises local temperature, and fever is suggestive of infective pre-patellar bursitis [8]. Clergyman’s knee or superficial infra-patellar bursitis may develop following repeated friction of the respective bursa between the skin and patellar tendon. Similarly, deep infra-patellar bursitis found in adolescents and presenting with focal anterior knee pain just below the lower pole of the patella that aggravates with extreme knee flexion and extension is another diagnostic confusion with OSD, though the absence of patellar tendon thickening favors the existence of previous clinical conditions [9]. Pes anserinus bursitis or anserine bursitis is due to the inflammation of the pes anserinus (anserine) bursa, along with its associated tendons, located on the proximo-medial aspect of the tibia. Though patients could present with isolated anserine bursitis, it has often coexisted with knee disorders, for example, osteoarthritis knee. Sporting activities, long-distance cycling/walking, diabetes, and obesity are also important risk factors for the condition [11-12]. Sometimes incorrectly, it could be documented as anterior knee pain.

The unique clinical features are sufficient for an OSD diagnosis. Alongside clinical manifestations, sometimes, radio imaging could be of great value while differentiating between different anterior knee pain syndromes. In most cases, the radiological finding is inconclusive in differentiating different anterior knee pain conditions, including that of soft tissue origin; however, a characteristic radio-opaque fragmented bone mass appearing over the tibial tuberosity is often enough for isolating OSD from other focal anterior knee pain disorders [3]. An X-ray could reveal a pre-patellar soft tissue swelling in pre-patellar and infra-patellar bursitis. Magnetic resonance imaging (MRI) could be effective in delineating different soft tissue lesions in and around the knee, for example, hyper-intense lesions before and after the patellar tendon, respectively, favor the clergyman’s knee and parson’s knee (infra-patellar bursitis) diagnosis. MRI changes in the patellar tendon are also seen in association with OSD, though not with infra-patellar bursitis [13]. Soft tissue swelling as of anserine bursa, pre-patellar bursa, etc. reveals low and high signal intensity, respectively, on MRI T1 and T2 scanning [13]. However, a routine approach for diagnosing bursitis under MRI should be restricted to when other approaches are inappropriate. The role of MRI in diagnosing OSD is currently limited and I did not perform it in the present case subject, as it seemed inappropriate to me; rather, I went with the traditional approach and further exploration was done with USG [2].

The high-frequency ultrasonogram (USG) could depict pathological changes in soft tissues and could play a pivotal role in differentiating OSD from other anterior knee pain disorders [1]. USG can detect every pathological change concerning OSD, including cartilage swelling, fragmentation of the tuberosity ossification center, patellar tendon lesions, and reactive bursitis; and based on these USG findings, it could be of three types: type 1, delamination of the internal ossification center; type 2, delamination tear or fracture of the epiphyseal part of the tibial tuberosity; and type 3, delamination tear of the ossification center resulting in an irregular deformation of the tuberosity [1]. And according to this grading, my presenting subject could be classified as type-3 OSD. However, in the disorder, USG may reveal a normal tubercle, and signal changes consistent with thickening (more echogenic) in the patellar tendon, as seen in MRI, could be the only presenting sign [13-14]. Ultrasonographic scanning has also the potential of differentiating OSD from jumper’s knee and Sinding-Larsen-Johansson syndrome [1]. In a recent meta-analysis, USG has been reported to have the potential of predicting future tendinopathy even in asymptomatic individuals [15].

Regarding treatment, the short-term use of NSAIDs could cause pain relief in OSD, however, it is not recommended to use steroids in this condition. In addition, activity limitation, the local application of ice, protective padding, and quadriceps/hamstring strengthening exercises provide further relief. Surgery to treat Osgood-Schlatter disease (OSD) is rarely recommended, especially in skeletally immature patients [2,16]. In this present study, the patient's sufferings were managed with conservative approaches: oral NSAIDs and abstinence from contact sports, with complete pain relief as documented at the three-month follow-up.

## Conclusions

Osgood-Schlatter disease is an anterior knee pain disorder, especially among physically active children. Besides clinical manifestations, unique radiological characteristics can clearly delineate the disorder from its mimicries. It appears that an ultrasonographic exploration could extend our understanding of the ailment, including pathological changes that are in progress with the Osgood-Schlatter disease, though further researches are warranted in addressing the issue.
